# The neural basis of task switching changes with skill acquisition

**DOI:** 10.3389/fnhum.2014.00339

**Published:** 2014-05-22

**Authors:** Koji Jimura, Fabienne Cazalis, Elena R. S. Stover, Russell A. Poldrack

**Affiliations:** ^1^Imaging Research Center, The University of Texas at AustinAustin, TX, USA; ^2^Precision and Intelligence Laboratory, Tokyo Institute of TechnologyYokohama, Japan; ^3^Department of Psychology, University of California Los AngelesLos Angeles, USA; ^4^Department of Psychology, The University of Texas at AustinAustin, TX, USA; ^5^Department of Neurobiology, The University of Texas at AustinAustin, TX, USA

**Keywords:** executive control, learning, procedural memory, multivariate pattern information, functional MRI

## Abstract

Learning novel skills involves reorganization and optimization of cognitive processing involving a broad network of brain regions. Previous work has shown asymmetric costs of switching to a well-trained task vs. a poorly-trained task, but the neural basis of these differential switch costs is unclear. The current study examined the neural signature of task switching in the context of acquisition of new skill. Human participants alternated randomly between a novel visual task (mirror-reversed word reading) and a highly practiced one (plain word reading), allowing the isolation of task switching and skill set maintenance. Two scan sessions were separated by 2 weeks, with behavioral training on the mirror reading task in between the two sessions. Broad cortical regions, including bilateral prefrontal, parietal, and extrastriate cortices, showed decreased activity associated with learning of the mirror reading skill. In contrast, learning to switch to the novel skill was associated with decreased activity in a focal subcortical region in the dorsal striatum. Switching to the highly practiced task was associated with a non-overlapping set of regions, suggesting substantial differences in the neural substrates of switching as a function of task skill. Searchlight multivariate pattern analysis also revealed that learning was associated with decreased pattern information for mirror vs. plain reading tasks in fronto-parietal regions. Inferior frontal junction and posterior parietal cortex showed a joint effect of univariate activation and pattern information. These results suggest distinct learning mechanisms task performance and executive control as a function of learning.

## Introduction

One of the hallmarks of skill acquisition is that a task that initially requires substantial executive resources can come to be performed in a manner that seems effortless (James, 1890). This behavioral facilitation has been characterized as a development of procedural or implicit memory (Roediger, 1990; Schacter et al., 1993; Gupta and Cohen, 2002) that is dissociable from declarative memory implicated in medial temporal structures (Cohen and Squire, 1980; Martone et al., 1984). Learning novel skills involves brain-wide reorganizations guiding optimal recruitments of cognitive functions (Salmon and Butters, 1995; Petersen et al., 1998; Dayan and Cohen, 2011). Importantly, any skill consists of a series of cognitive processes governed via executive control systems (Smith and Jonides, 1999; Miller and Cohen, 2001), which are decreasingly necessary as expertise is acquired (Milham et al., 2003; Kelley et al., 2006). However, it is unclear how executive control interacts with the acquisition of a novel skill.

Flexibility of behavior is a fundamental function of fronto-striatal pathways (Milner, 1963; Jones and Mishkin, 1972; Frank and Claus, 2006; Stelzel et al., 2010). This function has been examined in task-switching paradigms where different tasks are alternated (Jersild, 1927; Allport et al., 1994; Rogers and Monsell, 1995), in which response times are generally slower when switching tasks as opposed to repeatedly performing the same task (i.e. “switch cost”). Previous work has found evidence for asymmetric switch costs as a function of task difficulty (Yeung et al., 2006; see also Hikosaka and Isoda, 2010), and Yeung and Monsell (2003) showed that switch costs can be modulated by recent practice on a task. The degree to which these asymmetric switch costs are associated with different neural mechanisms is currently unknown.

**Figure 1:**
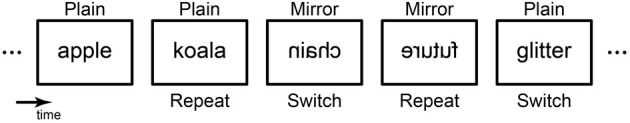
**Behavioral paradigm**. Participants performed a living/non-living semantic judgment task for visually presented words. In some trials, words are mirror-reversed, in which they had to read the words in a novel (untrained) way, whereas non-reversed canonical forms (plain) of words are presented in other trials. These trials can also be classified as to whether the current trial type were repeated or switched from the preceding trial. The switch trials require immediate and flexible change of task skill from one to another, whereas repeat trials do not. Participant performed the identical paradigm during pre- and post-training sessions.

To address these questions, the current fMRI study examined task switching in the context of learning a new cognitive skill. Participants unexpectedly alternated two tasks, one demanding a novel visual skill (mirror-reversed word reading), and the other involving a well-learned skill (plain word reading) (Figure 1; Kolers, 1968; Poldrack et al., 1998; Poldrack and Gabrieli, 2001; Pegado et al., 2011). They then received three behavioral training sessions on mirror reading over 2 weeks before the second fMRI session. A combination of univariate and multivariate fMRI analyses were used to examine the neural correlates of task switching over the course of training.

## Materials and methods

### Participants

Fourteen healthy human participants completed the study (mean age 22.4 years, range 19–35; 10 females). All volunteers gave informed consent according to procedures approved by the UCLA Office for Protection of Research Subjects. They were native English speakers, and right-handed as determined by the Edinburgh handedness inventory to ensure consistency of lateralized language representation without history of neuropsychiatric disorders or currently taking psychoactive medications.

### Behavioral procedures

Subjects took part in two MRI scanning sessions separated by 2 weeks; during the intervening period they received three training sessions on the mirror-reading task.

#### fMRI task

Participants performed living-non-living judgments on words that were presented in either plain or mirror-reversed text, across six fMRI scanning runs in each of the two sessions. The task was based on previous studies of skill acquisition in the mirror-reading task (Poldrack et al., 1998; Poldrack and Gabrieli, 2001) but modified such that plain and mirror-reversed trials were randomly intermixed, allowing the examination of task switching effects. On each trial, subjects were presented with a word and asked to decide whether the word named a living or non-living entity, and to press the corresponding button as quickly as possible (Figure 1). No warning was presented before the presentation of the word. Each run included 32 plain and 32 mirror-reversed words. There were a total of 12 word lists (6 runs in 2 sessions); order of presentation of the 12 word lists was counterbalanced across participants/sessions, and word length was equated within each list. This ensures that no words are repeated from the first to second training session, such that any learning effects reflect general skill rather than item-specific learning.

The timing and order of stimulus presentation was optimized for estimation efficiency using custom MATLAB code (Dale, 1999); the response window was 3.25 s, and the stimulus-onset asynchrony (SOA) varied across trials from 4 to 11.5 s (mean SOA = 6.28 s). These stimulus onset lists were also counterbalanced across runs over participants. Because the plain and mirror-reversed words were pseudorandomly presented, trials were split according to whether the stimulus condition presented immediately before the current trial was the same or different. This resulted in four types of trial: Mirror-Repeat (MR-RP), Mirror-Switch (MR-SW), Plain-Repeat (PL-RP), and Plain-Switch (PL-SW). Switching between the two stimulus types occurred on 34% of trials.

Stimulus presentation and timing of all stimuli and response events were achieved using the MATLAB Psychophysics Psychtoolbox (http://www.psychtoolbox.org/). Visual stimuli were presented using MRI-compatible goggles (Resonance Technologies, Van Nuys, CA), and the computer was synchronized with the onset of each functional run to ensure accuracy of event timing.

#### Training

Following the initial scan, participants participated in three behavioral training sessions, during each of which they were presented with 10 passages written entirely in mirror-reversed text. The participants were instructed to read the passages, each of which was several paragraphs long, as quickly as possible, and time taken to read each passage was recorded. After each passage, participants were given a multiple-choice question related to the content of the passage, to ensure reading for comprehension. The three training sessions were spaced over a period of 2 weeks, with no more than one session on any single day.

### Imaging procedures

Scanning was performed using a 3T Siemens (Erlangen, Germany) Allegra MRI scanner at the UCLA Ahmanson-Lovelace Brain Mapping Center. Functional images were acquired using T2^*^-weighted EPI [slice thickness: 4 mm; 30 slices; TR (repetition time) = 2 s; TE (echo time) = 30 ms; FA (flip angle) = 90°; matrix size: 64 × 64; FOV (field of view) = 200 mm]. Each functional run consisted of 205 functional volumes. For registration, a T2-weighted matched-bandwidth high-resolution anatomical scan (same slice prescription as EPI, but higher in-plane resolution) was acquired in both of the two sessions. Additionally, a magnetization-prepared rapid-acquisition gradient echo (MPRAGE) image was acquired for each participant in the first session (TR = 2.3; TE = 2.1; FOV = 256 mm; matrix size: 192 × 192; saggital plane; slice thickness = 1 mm; 160 slices).

### Preprocessing

Preprocessing was performed using the FSL suite (http://www.fmrib.ox.ac.uk/fsl/) (ver. 4.1.5). Brain extraction and motion correction were first performed for each of the functional runs. Functional images were then spatially smoothed using 5-mm full-width-half-maximum Gaussian kernel. For each functional run, registration was performed through a non-linear 3-step procedure implemented by FNIRT in FSL, whereby EPI images were first registered to slice-matched high-resolution T1 structural image, then to the high resolution MPRAGE structural image, and finally into 2 × 2 × 2-mm MNI standard space, using linear affine transformations by 12 parameters and non-linear displacement based on deformation fields.

### Univariate analysis

Voxel-wise GLM analysis was performed with FSL using a three-stage approach to implement a mixed effects model treating participants as a random effect. Individual functional runs were independently modeled at the first level. Four types of trial were modeled as effects of interest (MR-SW, MR-RP, PL-SW, and PL-RP). Each trial was coded by a delta function time-locked to the onset of the stimuli, convolved with the double-gamma canonical hemodynamic response function (HRF). Response times (RT) of individual trials were also included as a nuisance parametric modulation for each condition, convolved with the double-gamma canonical HRF. The RTs were mean-subtracted within each of the conditions before the convolution, and orthogonalized to the main effects. This procedure was intended to minimize general RT effect that occurred on trial-by-trial basis, but note that it does not remove effects that are correlated with RT differences between conditions. Six movement parameters were also included as nuisance effects. Temporal derivatives were included for all regressors.

Parameters were estimated using FILM after 64-s high-pass temporal filtering. A second-level analysis was then performed based on a fixed-effects model where all six functional runs per session were combined within each individual participant. Group-level statistics were then estimated based on *t*-tests for effects of interest. Finally, group level z-statistic images were thresholded using a uncorrected cluster-forming threshold of *Z* > 2.3 and a whole-brain corrected extent threshold of *p* < 0.05 based on Gaussian Random Field theory. Peak MNI coordinates above *Z* > 3.0 within the significant clusters are listed in the tables; if there were multiple peaks within 15 mm, the most significant was reported.

### Multivariate pattern analysis

#### Preprocessing

The identical data set was used as in the univariate analysis. The data were first realigned across the 12 functional runs (6 runs each in pre- and post-training sessions) to correct head movements during and across runs (whereas in the univariate analysis, each run was realigned only to itself, and then separately normalized to standard space). The reference volume was the mean image of the middle volumes across the runs that were aligned prior to the cross-run realignment. This procedure was intended to consistently realign functional volumes across all runs, since the MVPA analysis required combination of un-normalized data across runs. No spatial smoothing was applied to the EPI images.

The first-level analysis used the same GLM model as univariate analysis. Parameters were estimated using FILM after 64-s high-pass temporal filtering in native space without spatial smoothing. This estimation provided voxel-wise Z-maps for MR-SW, MR-RP, PL-SW, and PL-RP for each of the 12 functional runs (i.e., 6 pre- and 6 post-training sessions). Similarly to the univariate analysis, RT was modeled across the conditions in a separate analysis, and we confirmed this coding didn't change our main findings significantly.

#### Classification

Binary classification was performed using a searchlight procedure with a 3-voxel radius. A support vector machine with a linear kernel, as implemented in LibSVM (Chang and Lin, 2011) through PyMVPA (http://www.pymvpa.org/; Hanke et al., 2009), was used to classify trial types. Leave-one-out cross-validation was applied across the 6 functional runs in each session (pre- or post-training) in order to obtain the predicted classification for each left-out run. Training and test were performed within each of the pre- and post-training sessions.

Test and training signal data were normalized (i.e., mean subtracted out and then divided by standard deviation) within each region of interest (i.e., searchlight) (Misaki et al., 2010). Effects of the epsilon parameter in the SVM were evaluated by systematically testing the model with epsilon values from 0.0001 to 1 with by powers of 10; the results were consistent across these parameter values, and the current study reports the results with epsilon = 0.01.

#### Group analysis

Classification accuracy was contrasted between pre- and post-training sessions across the whole brain at the group level. This voxel-by-voxel subtraction was intended to test whether the regional pattern information of the trial types was changed through behavioral training on mirror reading. Accuracy maps of classification were first registered into MNI standard space using the same method as the univariate analysis for individual participants. The transformation parameters were estimated by FNIRT in FSL based on three-stage procedure as in the univariate analysis. Then, registered maps from all participants were subjected to a group-mean one-sample *t*-test based on permutation methods implemented the randomize tool in FSL (5000 permutations), and then thresholded using clusters determined by *Z* > 2.3. Each cluster was inspected for significance at *P* < 0.05 corrected for multiple comparisons for whole brain using the maximum statistic approach (Nichols and Holmes, 2002).

#### Empirical estimations of false positive rate

Because of potential bias in SVM results (c.f., Cohen et al., 2010; Jimura and Poldrack, 2012), we used randomization testing to estimate the distribution of classifier accuracy under the null hypothesis of no association between brain activity and the variable of interest. For each participant, trial condition labels were randomly shuffled within individual functional runs, and then the same SVM and group-level analysis was performed. This procedure was repeated 100 times. Then, group-level statistics from 100 randomizations were collected to test whether the identified regions in the original multivariate pattern analysis were above 95 percentile. The reported clusters in Table 3 all satisfied this criterion.

Because of the significant computational requirements of randomization with whole-brain searchlight analyses (more than 8000 processing hours), we conducted the analysis on the Ranger Linux Cluster (62976 computing cores) developed and maintained by Texas Advanced Computing Center (http://www.tacc.utexas.edu/).

### Conjunction of univariate and multivariate pattern analysis

A conjunction analysis was then performed in order to identify common brain regions that showed univariate and multivariate-pattern effects. In order to test for a significant conjunction compared to the conjunction null hypothesis (Nichols et al., 2005), binarized thresholded maps (*P* < 0.05 corrected for multiple comparisons for the whole brain) were multiplied in a voxel-wise manner between univariate analysis and MVPA and clusters with 8 or more continuous voxels are reported.

In order to more directly compare the univariate and multivariate analyses, we also performed a “univariate searchlight” analysis (Jimura and Poldrack, 2012). In this analysis, using the identical dataset of the MVPA, the mean levels of activation were calculated across voxels within identical searchlight space used in MVPA. Then, the mean univariate effect of learning [e.g., (MR-RP-PRE minus PL-RP-PRE) minus (MR-RP-POST minus PL-RP-POST)] was collected from all participants. Finally, group-level statistics were estimated to test if group effects were significant using the same procedure as in the MVPA.

## Results

### Behavioral results

**Figure 2:**
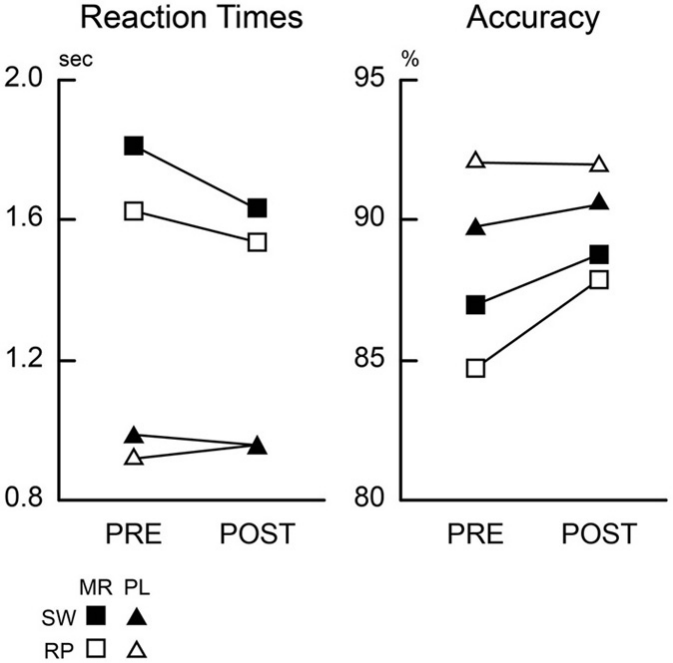
**Behavioral results**. Mean reaction times and accuracy across participants are plotted for pre-training (PRE) and post-training (POST) sessions. Black-filled squares and triangles indicate mirror switch (MR/SW) and plain switch (PL/SW) trials, respectively. Open squares and triangles indicate mirror repeat (MR/RP) and plain repeat (PL/RP), respectively.

In pre-training session, reaction times (RT) were modulated by the task types (mirror and plain reading) and switch of the tasks (switch and repeat trials) (Figure 2). A repeated measures Two-Way analysis of variances (ANOVA) with task and switch as factors revealed significant main effect of the task [*F*_(1, 13)_ = 194.3, *P* < 0.001], and task switch [*F*_(1, 13)_ = 28.1, *P* < 0.001], with a marginally significant interaction effect [*F*_(1, 13)_ = 3.64, *P* = 0.07]. *Post-hoc t*-tests revealed significant switch costs (differences between switch trials relative to repeat trials) in both of the mirror- and plain-reading conditions [Mirror: *t*_(13)_ = 3.46, *P* < 0.01; Plain *t*_(13)_ = 2.40, *P* < 0.05]. Further *post-hoc t*-tests revealed significant main effects of the task (Mirror vs. Plain) in both of the switch and repeat trials [Switch: *t*_(13)_ = 10.9, *P* < 0.001; Repeat: *t*_(13)_ = 12.5, *P* < 0.001]. These results suggest dissociable RT modulations specific to mirror and plain reading, as well as switch to these tasks. Accuracy was modulated by the task [*F*_(1, 13)_ = 10.2, *P* < 0.01], but not by the switch condition [*F*_(1, 13)_ = 0.14, *P* = 0.72].

Performance of the mirror-reading task increased across the three training sessions, with decreased paragraph reading times 244.8, 181.0, and 157.2 s in the first, second, and third practice sessions respectively [*F*_(2, 26)_ = 16.3, *P* < 0.001].

This training on the mirror-reading task resulted in improved performance on the judgment task for mirror-reversed items, demonstrating a skill transfer from the training task. A Three-Way repeated measures ANOVA on RTs with stimulus type (mirror, plain), switching (switch, repeat), and training (pre-, post-) as factors revealed significant interaction effects of training and task [*F*_(1, 13)_ = 12.6, *P* < 0.01], training and task switch [*F*_(1, 13)_ = 5.97, *P* < 0.05], switch and task [*F*_(1, 13)_ = 13.0, *P* < 0.01], along with main effects of task [*F*_(1, 13)_ = 120.5, *P* < 0.001] and task switch [*F*_(1, 13)_ = 82.9, *P* < 0.001]. *Post-hoc* repeated measures Two-Way ANOVAs on plain and mirror-reversed items (with training and task switch as factors) showed a significant main effect of training for mirror-reading [*F*_(1, 13)_ = 7.34, *P* < 0.05] but no effect for plain reading [*F*_(1, 13)_ = 0.59, *P* = 0.73], demonstrating that the effects of training were specific to mirror-reversed items. Accuracy of the mirror-reading task was improved accordingly [*F*_(1, 13)_ = 4.92, *P* < 0.05], although plain-reading performance was unchanged [*F*_(1, 13)_ = 0.02, *P* = 0.87].

### Imaging results

#### Univariate analysis

**Figure 3:**
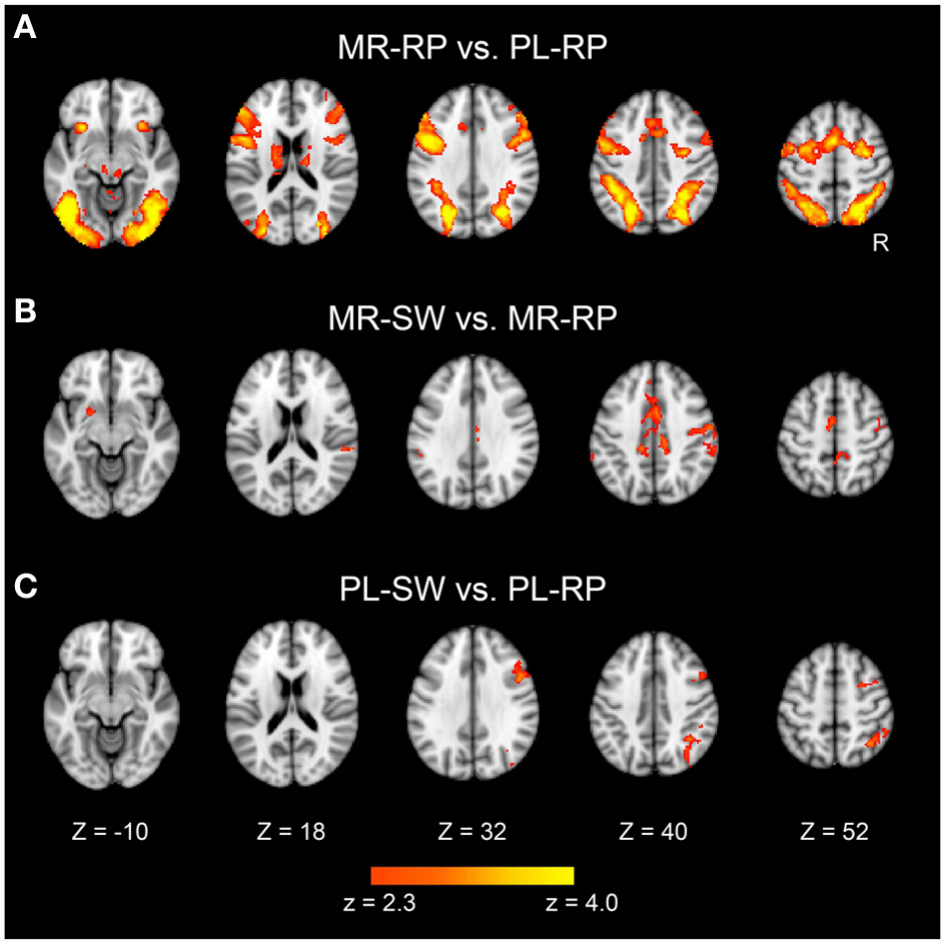
**Statistical significance maps for the contrasts Mirror-Repeat minus Plain-Repeat (A), Mirror-Switch minus Mirror-Repeat (B), and Plain-Switch minus Plain-Repeat (C) trials in the pre-training session (*P* < 0.05 cluster size corrected)**. The color scale reflects statistical significance as shown by the color bar to the bottom (above *Z* > 2.3 uncorrected). Maps are displayed as transverse sections and are overlaid on the top of the standard anatomical image. The levels of sections are indicated by the Z coordinates of MNI space. R, Right.

We first identified brain regions that were significantly activated during mirror reading relative to plain reading (Figure 3A). These regions included broad cortical areas across the brain, including inferior, middle, and superior frontal gyri, anterior insula, posterior and superior parietal cortices, and extrastriate cortices bilaterally. The region also included subcortical regions, including bilateral thalamus and caudate. These activations are consistent with prior studies of mirror reading using blocked designs (Poldrack et al., 1998; Poldrack and Gabrieli, 2001).

Next, the effects of switching between plain and mirror reading were explored. As shown in Figure 3B and Table 1, switching from plain text to mirror reading resulted in significant increases in activations in medial wall areas (pre-supplementary motor area, posterior dorsal part of anterior cingulate cortex), superior and posterior parietal cortices, and caudate, consistent with prior studies of task switching (e.g., Kimberg et al., 2000; MacDonald et al., 2000; Rushworth et al., 2002; Braver et al., 2003; Sakai and Passingham, 2003, 2006; Crone et al., 2006; Kim et al., 2011, 2012). Additionally, robust effects were observed in left dorsal striatum (putamen) and bilateral temporo-parietal junctions, the regions less common to conventional task switching paradigms (Figure 3B and Table 1). In contrast, switching from mirror-reading to plain reading showed increased activations in inferior frontal junctions and posterior and superior parietal cortices (Figure 3C and Table 1), regions commonly reported in previous literature of task switching (e.g., Dove et al., 2000; Kimberg et al., 2000; MacDonald et al., 2000; Braver et al., 2003; Koechlin et al., 2003; Sakai and Passingham, 2003, 2006; Brass and von Cramon, 2004; Crone et al., 2006; Jimura and Braver, 2009; Kim et al., 2011, 2012). Interesting, there was no overlap between regions engaged by switching from mirror-reading and those engaged by switching to mirror-reading.

**Table 1:** **Brain regions showing significant activations during switch trials relative to repeat trials in pre-training session**.

**Contrast**	**Region**	***x***	***y***	***z***	***z*-values**
MR-SW vs.	Right temporo-parietal junction	52	−32	22	3.80
MR-RP	Anterior cingulate	4	−12	30	3.53
	Right superior parietal	14	−54	62	3.38
	Posterior cingulate	8	−40	24	3.35
	Anterior cingulate/medial prefrontal	4	4	38	3.31
	Left temporo-parietal junction	−54	−40	32	3.25
	Left putamen	−20	4	−12	3.23
	Left caudate	−8	4	8	3.19
	Right temporo-parietal junction	64	−34	12	3.15
	Posterior cingulate	−12	−32	38	3.14
	Pre-supplementary motor area	−4	−4	50	3.12
	Superior temporal	−60	−26	8	3.12
	Right pre-central gyrus	50	−16	36	3.11
PL-SW vs.	Lingual gyrus	6	−68	8	3.53
PL-RP	Right superior parietal	36	−58	52	3.46
	Right posterior parietal	36	−52	38	3.37
	Right inferior frontal junction	48	8	32	3.25
	Lingual gyrus	−12	−82	2	3.21
	Right superior frontal	40	2	56	3.16

The regions are listed in the order of z-value. MR-SW, mirror switch; MR-RP, mirror repeat; PL-SW, plain switch; PL-RP, plain repeat.

**Figure 4:**
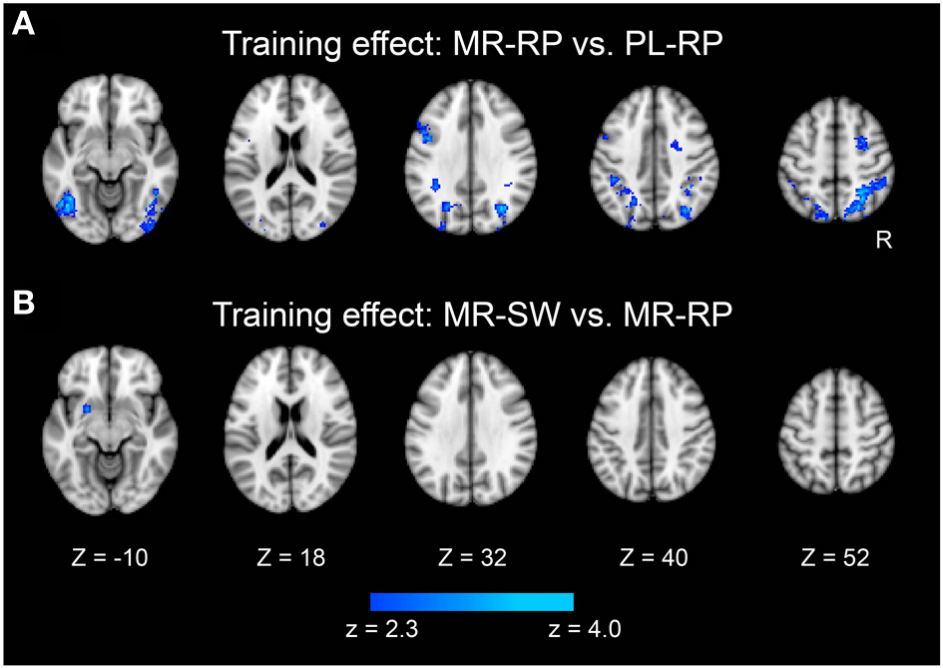
**Statistical significance maps for training-related decreases in the contrasts Mirror-Repeat minus Plain-Repeat (A) and Mirror-Switch minus Mirror-Repeat (B) (*P* < 0.05 cluster size corrected)**. The formats are similar to those in Figure 3.

Training effects of mirror reading were examined by comparing pre-training and post-training activation. As shown in Figure 4A and Table 2, multiple frontal, parietal, temporal regions showed decreased activation in the post-training session relative to pre-training session, consistent with prior work (Poldrack et al., 1998; Poldrack and Gabrieli, 2001). There were no regions that showed increased activity from pre-training to post-training.

**Table 2:** **Brain regions showing significant univariate activation decrease after behavioral training**.

**Contrast**	**Region**	***x***	***y***	***z***	***z*-value**
MR-RP vs. PL-RP	Left extrastriate	−42	−70	−8	4.19
	Right posterior parietal	34	−70	38	4.12
	Right extrastriate	48	−54	−14	3.98
	Right superior parietal	28	−58	52	3.89
	Left superior parietal	−34	−62	60	3.85
	Left posterior parietal	−12	−78	52	3.82
	Right lateral occipital	38	−82	10	3.77
	Right superior frontal	32	0	60	3.74
	Left inferior frontal	−46	6	30	3.69
	Left posterior parietal	−30	−52	46	3.62
	Left posterior parietal	−24	−68	48	3.58
	Left posterior parietal	−26	−84	36	3.57
	Left superior parietal	12	−72	50	3.54
	Left intra-parietal sulcus	−40	−38	36	3.53
	Left lateral occipital	−32	−88	12	3.50
	Right superior frontal	24	2	46	3.46
	Right extrastriate	54	−72	−2	3.45
	Right superior parietal	42	−44	50	3.45
	Right extrastriate	36	−92	−2	3.43
	Left lateral occipital	−28	−82	20	3.29
	Left posterior parietal	−24	−66	32	3.24
	Right extrastriate	46	−80	−18	3.12
MR-SW vs. MR-RP	Left putamen	−22	6	−10	3.24

The regions are listed in the order of z-value. MR-SW, mirror switch; MR-RP, mirror repeat; PL-RP, plain repeat.

We also examined how training altered the neural activity associated with task switching. A focal region in dorsal striatum (putamen) showed a significant decrease in switching-related activity between pre- and post-training (Figure 4B and Table 2). It is important that this dorsal striatum region also activated in the mirror-switch trial in the pre-training session (Figure 3B and Table 1), suggesting a training-related decrease in activation specific to switching to mirror reading. There were no training-related activation changes for switching to plain reading from mirror reading.

### Multivariate pattern analysis

**Figure 5:**
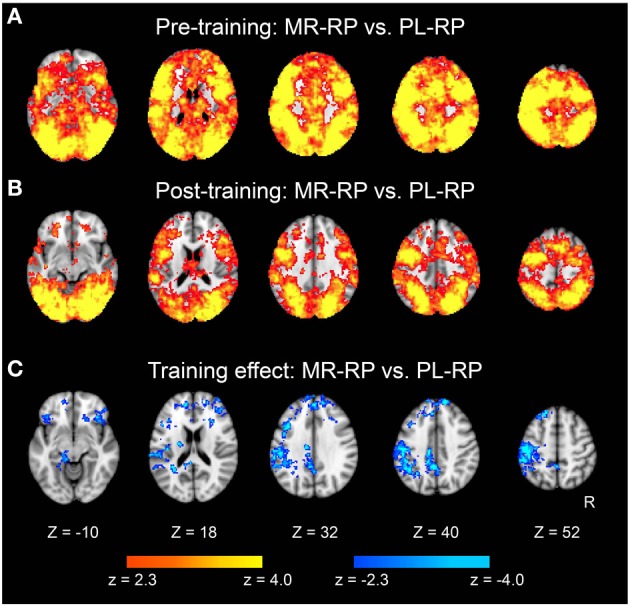
**Statistical significance maps for searchlight MVPA for Mirror-Repeat vs. Plain-Repeat trials (*P* < 0.05 cluster size corrected)**. **(A)** Pre-training session. **(B)** Post-training session. **(C)** The difference in classification accuracy between pre- and post-training session. Cool color indicates decreased accuracy in post-training session. The formats are similar to those in Figures 3, 4.

We then examined whether pattern information associated with the mirror-reading task changed with training. A searchlight multivariate pattern analysis (MVPA) was first performed for MR-RP and PL-RP trials within each of the sessions (see also Methods). As shown in Figure 5A, most gray-matter regions across the brain showed significant above-chance classification performance in the pre-training session. In the post-training session, many brain regions still showed significant accuracy, but the extent of voxels showing significant classification was decreased (Figure 5B). This decrease was significant in a direct comparison between pre- and post-training, primarily in frontal and parietal cortices (Figure 5C and Table 3). No regions showed increased classification accuracy from pre- to post-training. These results indicate that even after training broad areas still maintain regional pattern information that can discriminate mirror reading and plain reading, but the amount of information decreased in fronto-parietal regions.

**Table 3:** **Brain regions that showed significant decrease in classification performance in MVPA after behavioral training**.

**Contrast**	**Region**	***x***	***y***	***z***	***z*-value**
MR-RP vs. PL-RP	Left lateral occipitotemporal	−32	−46	20	4.81
	Right superior frontal	14	52	40	4.80
	Pre-cuneus	−4	−46	36	4.61
	Left post-central gyrus	−48	−22	52	4.52
	Medial orbital cortex	18	28	−22	4.49
	Pre-central gyrus	−6	−30	68	4.40
	Right inferior frontal	44	34	6	4.36
	Left supramarginal gyrus	−52	−26	30	4.32
	Left superior frontal	−20	34	54	4.10
	Left posterior parietal	−42	−50	48	4.06
	Left anterior insula	−34	28	4	4.03
	Medial pre-frontal	0	50	34	3.87
PL-SW vs. PL-RP	Left pre-central sulcus	−62	2	34	4.56
	Left central sulcus	−54	−22	26	4.56
	Left anterior temporal	−46	−6	−28	4.38
	Left posterior parietal	−46	−38	48	4.32
	Right inferior temporal	60	−4	−26	3.93
	Right inferior temporal	24	0	−42	3.90
	Right ventral pre-frontal	52	28	−8	3.80
	Right anterior insula	44	2	−8	3.79
	Left inferior pre-frontal	−58	−2	16	3.79
	Right inferior pre-frontal	50	18	24	3.67
	Right anterior insula	20	26	−6	3.65
	Left posterior parietal	−38	−60	40	3.63

The regions are listed in the order of z-value. MR-RP, mirror repeat; PL-RP, plain repeat; PL-SW, plain switch.

**Figure 6:**
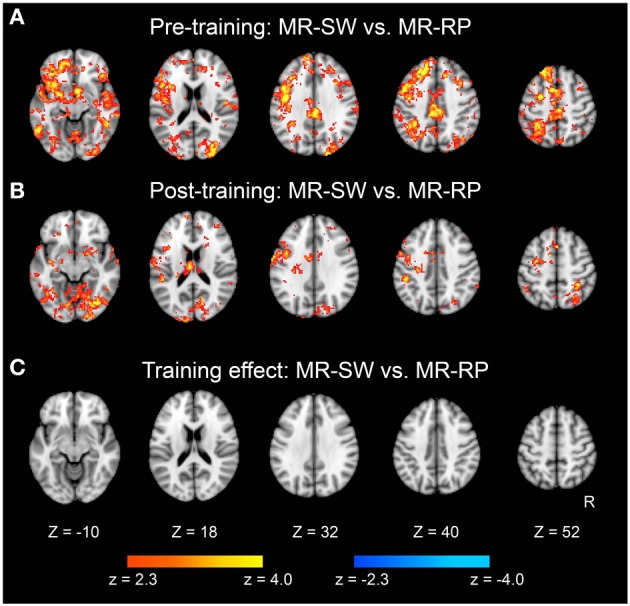
**Statistical significance maps for searchlight MVPA for Mirror-Switch vs. Mirror-Repeat trials (*P* < 0.05 cluster size corrected). (A)** Pre-training session. **(B)** Post-training session. **(C)** The difference in classification accuracy between pre- and post-training session. The formats are similar to those in Figure 5.

**Figure 7:**
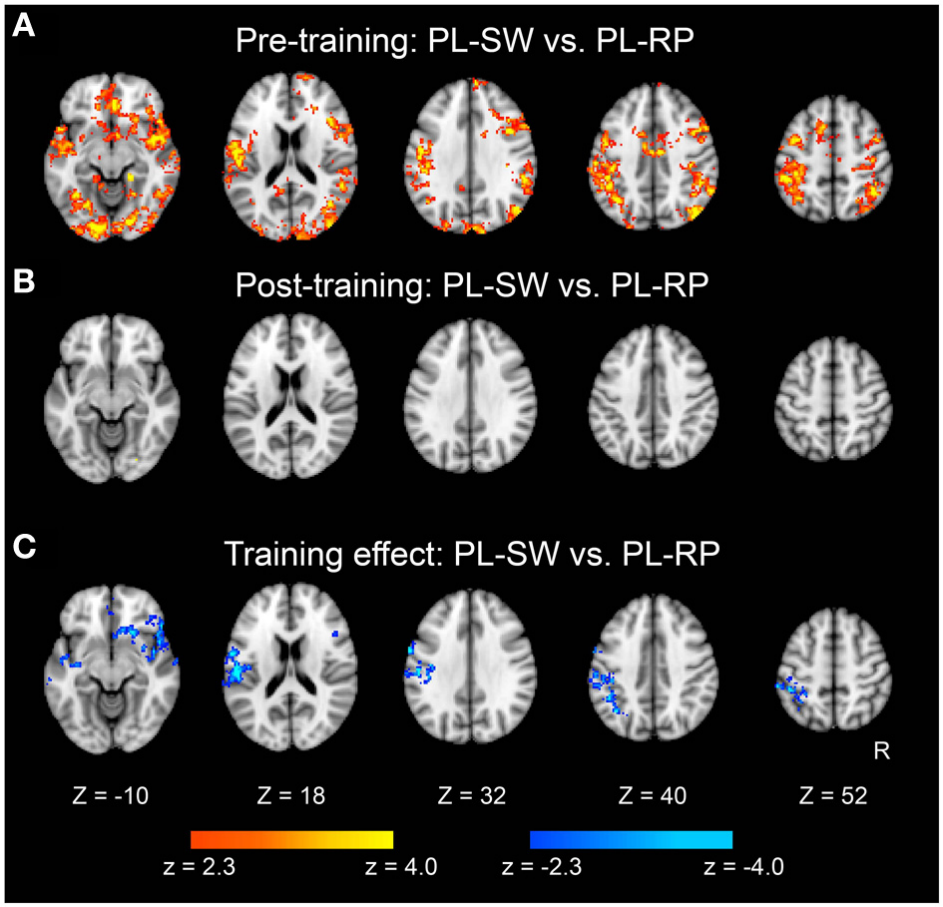
**Statistical significance maps for searchlight MVPA for Plain-Switch vs. Plain-Repeat trials (*P* < 0.05 cluster size corrected). (A)** Pre-training session. **(B)** Post-training session. **(C)** The difference in classification accuracy between pre- and post-training session. The formats are similar to those in Figures 5, 6.

We also examined classification of switch vs. non-switch trials. As shown in Figure 6A, brain regions showed significant classification accuracy for mirror reading switch trials (i.e., MR-SW and MR-RP) in the pre-training session. The extent of classification accuracy was visually reduced in the post-training session (Figure 6B), but the difference was not significant by direct comparisons (Figure 6C). Switching to plain reading from mirror reading also revealed smaller regions in the pre-training session (Figure 7A). However, even lesser region showed significant effects in the post-training session (Figure 7B). Indeed, the training-related comparison of classification accuracy did reveal right fronto-temporal and left fronto-parietal regions showing significant decrease in classification accuracy in the post-training session (Figure 7C and Table 3).

**Figure 8:**
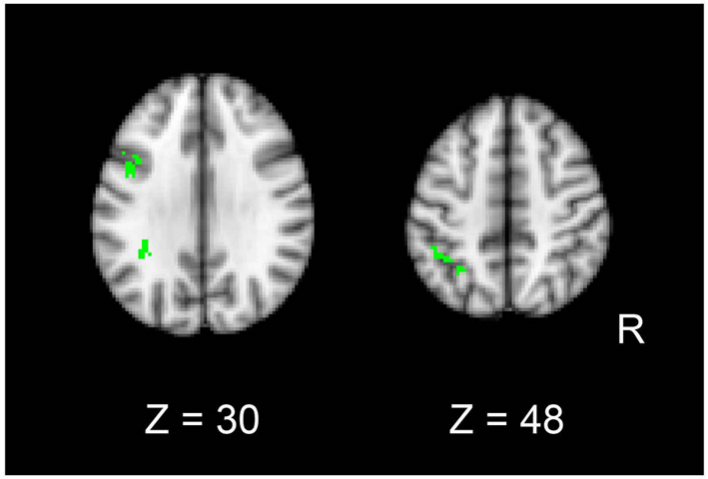
**Conjunction maps of the univariate analysis and MVPA for the contrast Mirror-Repeat vs. Plain-Repeat**.

In order to identify common signals between univariate and MVPA analyses, we performed a conjunction analysis. Fronto-parietal regions showed significant decreases in both pattern information and univariate activation for mirror reading (i.e., MR-RP vs. PL-RP; conjunction of Figures 4A, 5C). The regions included inferior frontal junction and posterior parietal cortex in the left hemisphere (Figure 8 and Table 4). Thus, in these regions the training of mirror reading decreased both local univariate activity and discriminable voxel pattern information in MR-RP and PL-RP trials. Because the spatial characteristics were different between the two analyses, we performed a follow-up “searchlight univariate analysis” in which the same spatial exploration was used as in MVPA (see also Methods; Jimura and Poldrack, 2012). This analysis confirmed this conjunction effect in the inferior frontal junction and posterior parietal cortex, ensuring that the current conjunction effects may not be attributable to different spatial characteristics between the standard univariate analysis and MVPA.

**Table 4:** **Brain regions that showed conjunction effects of decreasing univariate activity and classification performance**.

**Contrast**	**Region**	***x***	***y***	***z***	**Cluster extent**
MR-RP vs.	Left supramarginal gyrus	−39.5	−42.9	39.5	160
PL-RP	Left inferior frontal junction	−42.5	9.5	29.7	80
	Superior parietal	−28.1	−55.0	46.1	20

The regions are listed in the order of cluster extent size (voxel). MR-RP, mirror repeat; PL-RP, plain repeat.

## Discussion

The current study examined task switching in the context of acquisition of novel visuospatial skill. Training on the mirror-reading task led to decreased response times as well as decreased cost of switching from plain reading to the mirror-reading task. Neurally there was a widespread decrease in both activation and pattern information from pre-training to post-training for mirror-reversed compared to plain text items; no significant increases were observed. Non-overlapping patterns of switching-related activation were seen for the mirror-reading and plain-text tasks; learning was associated with decreased switching-related activation for mirror-reversed trials in the putamen, and for decreased switching-related pattern information in right pre-frontal and left parietal regions. A conjunction of activation and MVPA analyses showed joint effects of training on activation and information in the inferior frontal junction and posterior parietal cortex, highlighting the consistency of these changes.

While previous work has examined the behavioral effects of switching between tasks that differ in difficulty (Yeung and Monsell, 2003), the degree to which they involve different neural systems has been unknown. The present results demonstrate that switching from an easy task to a difficult task is associated with a very different pattern of activation compared to switching from a difficult task to an easy task, and that these patterns are modulated by training. Switching from mirror reading to plain text was associated with activations in the inferior frontal junction (IFJ) and posterior parietal cortex; the IFJ in particular has been implicated in the updating of task representations (Brass and von Cramon, 2004; Derrfuss et al., 2004), which would be necessary when switching from the difficult task to the easy task. Conversely, switching from plain text to mirror-reading engaged a large set of regions in the medial wall (including anterior cingulate and pre-SMA) along with the striatum and right parietal cortex. We propose that these regions register the need to switch from the highly-practiced task and exert the control necessary to engage the novel task set.

Previous work (Poldrack et al., 1998; Poldrack and Gabrieli, 2001) demonstrated increased activation in the inferior temporal cortex associated with training on the mirror-reading task, whereas no increases in activation or pattern information were observed in the present study. While this may reflect a lack of power, it could also reflect differences in the training procedures used in the studies. In the previous studies, subjects trained on the same mirror-reading task used during scanning (lexical decision), whereas in the present study subjects performed a living-non-living task in the scanner while the training involved reading of paragraphs of mirror-reversed text. The behavioral improvements observed in this study show that the paragraph training procedure was effective at improving mirror-reading skill on the task used during scanning, but it may be the case that increases in activation (at least for the amount of training in this study) require greater overlap of training and test tasks. Given the substantial current interest in the generalization of training, this could be a fruitful avenue for further exploration.

While many previous studies have used univariate activation analyses to examine learning-related changes, we are not aware of any that have used MVPA approaches to examine how pattern-information changes with learning of cognitive skills. The present analyses suggest that pattern-information analyses are much more sensitive to task-related differences as well as to learning-related changes, compared to univariate approaches. This is consistent with the results of previous analyses showing substantially greater sensitivity of MVPA approaches (Jimura and Poldrack, 2012). The source of these differences remains unclear. In the present MVPA analyses, the mean activation across the searchlight was removed in order to focus on distributed pattern information. However, recent work (Davis et al., 2014) has shown that such analyses may still be sensitive to univariate activation effects when those effects vary across voxels within a searchlight, which is highly likely to occur. Thus, we are reticent to make strong claims that the different between MVPA and univariate signals are reflective of different aspects of neural or cognitive function.

## Author contributions

Koji Jimura, Elena R. S. Stover, Fabienne Cazalis, and Russell A. Poldrack designed and conceived the study and analyses. Elena R. S. Stover and Fabienne Cazalis collected the data. Koji Jimura and Russell A. Poldrack analyzed the data and wrote the manuscript. Elena R. S. Stover and Fabienne Cazalis commented on the manuscript.

### Conflict of interest statement

The authors declare that the research was conducted in the absence of any commercial or financial relationships that could be construed as a potential conflict of interest.
